# Preparation of Mineral Admixture from Iron Tailings with Steel Slag-Desulfurization Ash and Its Application to Concrete

**DOI:** 10.3390/ma15155162

**Published:** 2022-07-25

**Authors:** Yannian Zhang, Mengwei Dong, Wenjie Zhang, Hao Chen, Daokui Yang

**Affiliations:** School of Civil Engineering, Shenyang Jianzhu University, Shenyang 110168, China; zyntiger@163.com (Y.Z.); dmwymeng@163.com (M.D.); wenjiezhang1996@163.com (W.Z.); ch961113@126.com (H.C.)

**Keywords:** iron tailings, steel slag, desulfurization ash, admixture, chemical activation, concrete, compressive strength, mechanism of action

## Abstract

Iron tailing solid waste not only has a high annual output but also has a low comprehensive utilization rate. Low utilization rate of iron tailings seriously restricts the development of comprehensive utilization of solid waste. In order to prepare an iron tailings-based ternary solid waste admixture and to verify its application to concrete, first, the effect of solid waste synergy on the strength of an iron tailings-steel slag-desulfurization ash admixture (ISD) system was investigated. Second, the effect of chemical activator dosing on the strength of an ISD system was studied and the mechanism of chemical activator action on the ISD system was investigated by thermogravimetric analysis (TG-DTA) Then, the effect of this admixture on the strength of concrete was studied. Finally, the mechanism of the effect of this admixture on the strength of concrete was clarified by mercury intrusion porosimetry (MIP) and backscattering electron tests (BSE). The results showed that the 7 d and 28 d compressive strengths of the ISD admixture were significantly higher than those of iron tailings single admixture. The 7 d and 28 d compressive strengths of the ISD system reached 24.9 MPa and 36.1 Mpa, respectively, when the ratio of iron tailings:steel slag:desulfurization ash = 1:1:1. Na_2_SiO_3_ is suitable for the early strength agent of the ISD admixture, but the amount of admixture should not exceed 0.6% of the admixture. TG-DTA shows that Na_2_SiO_3_ is enhancing the early strength of the ISD system by promoting the consumption of Ca(OH)_2_ in the ISD system to produce C-S-H. However, in the late reaction of the ISD system, Na_2_SiO_3_ inhibits the late strength development of the ISD system by suppressing Ca(OH)_2_ production. Concrete with ISD dosing of 30% or less meets the C40 requirement. MIP and BSE show that ISD provides a filling effect to concrete, but also causes a reduction in the active reactants of concrete and the combined effect of microfilling and active effects affects the strength development of ISD concrete. This study provides a theoretical and scientific basis for the preparation of iron tailings-based ternary solid waste dopants, and, in addition, the study promotes the consumption of iron tailings solid waste and the development of multiple solid waste dopants.

## 1. Introduction

In recent years, the massive accumulation of solid waste, such as iron tailings and red mud, has caused serious problems to the ecological environment [1,2,3]. Studies related to iron tailings have described that the annual production of iron tailings in China is 47.5 million tons [4]. However, the annual comprehensive utilization rate of iron tailings is only 18.9%, and its utilization rate is far below the minimum standard of 20% in China [5]. Improving the utilization rate of iron tailings to reduce the accumulation of iron tailings is a current research hotspot. In order to make the best use of iron tailings, researchers have made attempts to apply iron tailings in different fields. For example, Jiang et al., found that iron tailings can be used as road base material by investigating the static and dynamic properties of fiber-modified lime and fly ash stabilized iron tailings under dry and wet cycles [6].The studies of Lv et al., on the comprehensive performance of iron tailing aggregate concrete [7] and Feng et al., on the mechanical properties of iron tailing aggregate concrete interface transition zones [8] both showed that iron tailing aggregate concrete has excellent mechanical properties. Tang et al.’s study on the preparation of concrete admixtures using iron tailings [9] and Han et al.’s study on precast concrete with iron tailing powder [10] both prepared iron tailing admixture concrete. The above scholars’ studies confirmed that iron tailings can be used as road base materials, concrete admixtures, and concrete aggregates. Regarding the application of iron tailings in the field of concrete admixtures, many scholars have conducted more in-depth studies. For example, research in the direction of durability and workability of concrete for iron tailings. Cheng et al., studied the durability of iron tailings admixture concrete and found that iron tailings can improve the permeability of concrete [11]. Wu et al., studied the effect of iron tailing powder and slag powder composite admixture on the performance of concrete and found that iron tailing can partially replace mineral powder as concrete admixture, which is conducive to improving the compatibility [12]. Wu et al., studied the effect of a composite admixture of iron tailing powder and slag powder on the frost resistance of concrete and found that iron tailing can optimize the pore structure of concrete and improve the frost resistance [13]. Han et al., studied the effect of iron tailings on the performance of fly ash concrete and found that iron tailings are beneficial to improving the early strength of concrete but reduce the later strength and improve the durability of concrete [14]. Secondly, many scholars have also conducted research in the direction of mechanical properties of iron tailings concrete. Liu et al., studied the effects of dolomite powder and iron tailing powder on the resistivity, strength and microstructure of cement paste and concrete, and found that the strength of concrete increased and then decreased with the increase in iron tailing powder admixture [15]. Lu et al., investigated the effect of curing conditions on the mechanical and microstructural properties of ultra-high-performance concrete incorporating iron tailing powder and found that the strength of concrete first increased and then decreased with the increase in iron tailing powder dosing [16]. Ling et al., conducted an environmental design study of ultra-high performance concrete using iron ore tailings as cement material and found that iron tailings promote optimization of long-term compressive strength and pore structure of concrete [17]. Another study on the influence of iron ore tailings concrete by working environmental conditions has been explored by several scholars, one after another. Han et al., studied the effect of different curing conditions on the performance of iron tailings concrete, and it was found that reducing the water–cement ratio or adding slag could improve the performance of concrete [18]. Han et al., investigated the effect of water–cement ratio and temperature on the heat of hydration and properties of slag iron tailing powder ternary blended cement and found that early high-temperature curing was beneficial to the properties of iron tailing powder and slag ternary cement [19]. Iron tailing admixture can improve the performance of concrete, has a good filling effect, and has a good application prospect in the direction of concrete admixture.

In addition, many scholars have conducted research not only on concrete properties, but also on the direction of iron tailings activation. Gu et al., studied the effect of mechanical activation and iron tailings content on ultra-high-performance concrete, and it was found that mechanically activated iron tailings are suitable for concrete admixtures [20]. Yao et al., studied the effect of mechanical activation on the potential volcanic ash activity and hydration properties of iron tailings and found that the mechanical properties of cement mixed with 10%, 20% and 30% activated iron tailings meet the requirements of 32.5 composite silicate cement [21]. Yao et al., investigated the volcanic ash activity of iron tailings by mechanical grinding and found that mechanically ground iron tailings are suitable as cement admixtures [22]. Many scholars have also studied the effect of various solid waste admixtures on iron tailings admixtures. Han et al., found that the addition of gypsum compensated for the early strength of the admixture by investigating the effect of gypsum on the performance of highly dosed slag iron tailings composite admixture [23]. Zhang et al., investigated the preparation of mine backfill from steel slag combined with ultrafine tailings and found that steel slag provides alkaline conditions favorable to the reaction of active minerals from ultrafine tailings [24]. Iron tailings can significantly improve the performance of the admixture by compounding other materials. Therefore, developing more iron tailings composite dopants will not only promote the utilization of iron tailings, but also the consumption of other solid wastes.

As a by-product of steelmaking, the annual production of steel slag in China amounts to more than 100 million tons [25]. Steel slag has more C_3_S and C_2_S in it, which has potential cementing activity [26]. However, the utilization rate of steel slag in China is only about 22% [27]. The utilization rate of steel slag needs to be further improved.

Desulfurization ash, a semi-dry flue gas desulfurization product, has an annual production of several billion tons in China [28]. Desulfurization ash is a typical calcareous waste, which is rich in Ca(OH)_2_ and other unstable components, limiting its resourcefulness [29].

In conclusion, it is potentially feasible to apply iron tailings, steel slag and desulfurization ash to composite admixtures. Systematic studies to prepare an ISD admixture, to investigate the effect of chemical activators on the strength of an ISD admixture and to verify the feasibility of an ISD admixture applied to concrete have not been carried out in depth, hindering the development of ISD systems in the direction of admixture and limiting the utilization rate of iron tailings.

The objective of this paper is to prepare a cement admixture from iron tailings–steel slag–desulfurization ash ternary solid waste. Firstly, the synergistic effect of the ternary solid waste was investigated by adjusting the ratio of each solid waste in the ISD system and determining the suitable ratio. Secondly, the effect of Na_2_SiO_3_ activator on the mechanical properties of the ISD admixture system was investigated by adjusting the Na_2_SiO_3_ activator dose on the basis of the suitable ratio and the mechanism of the effect of Na_2_SiO_3_ activator on the mechanical properties of the ISD admixture system was investigated by means of TG-DTA microscopic techniques. Finally, the ISD cement admixture was applied to concrete to verify its feasibility and analyze its mechanism of action by microscopic means.

## 2. Experiment

### 2.1. Raw Materials

Iron tailing powder: iron tailing powder from Crooked Head Mountain, Benxi, China. Steel slag: steel slag from Shanghai Baowu Iron and Steel Group, China. Desulfurization ash: desulfurization ash from Aoda Refractories Processing Plant, Lingshou County, Shijiazhuang City, China. Cement: P·O 42.5 benchmark cement from Shanshui Group, China. Activator: the Na_2_SiO_3_ produced by Tianjin Science and Trade was used as the chemical activator. Standard sand: China ISO standard sand produced by Xiamen Aisiou Standard Sand Co, China. Water: tap water is taken. Iron tailing waste rock and iron tailing sand are provided by Liaoning One Cube Sand Industry Co, China. Water reducing agent: P-II type air-entraining water reducing agent produced by Shenyang Sheng Xinyuan Building Materials Co, China.

**Figure 1:**
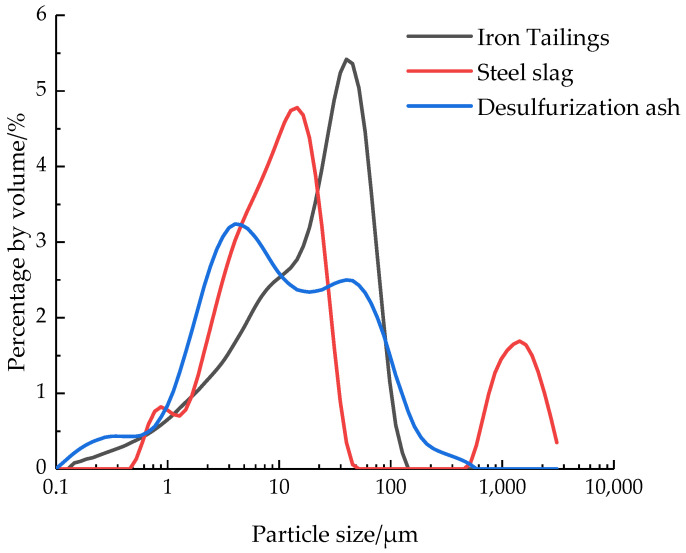
Particle size distribution of iron tailings, steel slag and desulfurization ash.

The chemical compositions and mass fractions of iron tailings, steel slag and desulfurization ash determined by X-ray fluorescence (XRF) are shown in Table 1. The particle size distribution curves of iron tailings, steel slag and desulfurization ash by laser particle size analyzer are shown in Figure 1.

### 2.2. Specimen Preparation

#### 2.2.1. Preparation of Mortar Specimen

This test is designed to replace 30% of cement with solid waste dopants, such as iron tailings. Referring to the “Test Method for Cementitious Sand Strength (ISO Method)” (GB/T17671-1999) and the specific test ratios shown in Table 2 and Table 3, according to the dopant-to-cement ratio 3:7, binder-to-sand ratio 1:3, water-to-binder ratio 0.5, weighing dopant, cement, standard sand and water, and the water, standard sand and dopant-to-cement were put into the JJ-5 planetary type mixer to prepare colloidal sand. The colloidal sand was put into a 40 mm × 40 mm × 160 mm triplex type steel mold to prepare the prismatic test block by vibrating table, then the test block was put under standard condition (temperature is 20 ± 1 °C, relative humidity is not less than 90%) for 24 h, the test block taken out of the mold and put into water under standard condition for 7 d, 28 d.

#### 2.2.2. Preparation of Concrete Specimen

Referring to the experimental proportioning scheme shown in Table 4, each admixture, cement and other experimental materials were weighed, and the weighed coarse and fine aggregates were poured into the concrete mixer and mixed for 1 min, then cement and admixtures were added and mixed for 1 min, and finally water and water-reducing agent were added and mixed for 2 min, and the concrete was poured into a 100 mm × 100 mm × 100 mm mold to prepare cubic specimens through a vibrating table, and the specimens were cured for 24 h under standard conditions (temperature of 20 ± 1 °C and relative humidity of not less than 90%), and the specimens were demolded and placed in water under standard conditions until 7 d, 14 d and 28 d.

### 2.3. Test Method

The compressive strength and flexural strength of the admixture specimens were determined concerning the “Test Method for Cementitious Sand Strength (ISO Method)” (GB/T17671-1999), and the average value of three parallel values was taken for each group of test results.

The compressive strength test of concrete specimens was carried out with reference to the standard for “Test Methods of Physical and Mechanical Properties of Concrete” (GB/T50081-2019). First, the 7 d, 14 d and 28 d compressive strengths of the concrete were tested using a Shenzhen brand universal testing machine (2000 kN) with a loading rate of 0.7 MPa/s. The compressive strength results are then converted using the conversion factor, which is taken as 0.95. The average value of compressive strength of three specimens was taken as the test result.

The gel water and Ca(OH)_2_ contents of 7 d and 28 d admixture specimens were determined by means of TG-DTA microanalysis. The specimens were heated to 812 °C for thermogravimetric analysis in a nitrogen atmosphere using a comprehensive thermal analyzer with a heating rate of 20 °C/min. The gel water and Ca(OH)_2_ contents at each hydration age were calculated from the following Equations (1) and (2):

(1) $$H_{2}O\left(\mathrm{Gel}\;\mathrm{water}\right)=E\times 0.01g$$

(2) $$\mathrm{Ca}{\left(\mathrm{OH}\right)}_{2}=F\times 0.01g\times \frac{74}{18}$$

where: E is the percent thermal weight loss caused by the dehydration of the gel; F is the percent thermal weight loss of water lost by the decomposition of Ca(OH)_2_; and where 18, 74 are the molecular weights of H_2_O and Ca(OH)_2_, respectively.

The concrete test samples were analyzed for mercury compression by mercury intrusion porosimetry (MIP) testing. First, the concrete pieces were cut parallel to the surface of the specimen using a cutter at a position 15 mm from the surface of the concrete block. Then, core samples (without drilling for aggregate) were drilled on the cut surface of the concrete test blocks using an electric drill and hollow core bit (8–14 mm inner diameter of the drill bit). After sampling, the samples were soaked in anhydrous ethanol for 7 days to terminate hydration, then dried for 3 days and tested for mercury compression (drying temperature of 50 ± 2 °C). Porosity and pore size distribution of the samples were measured at a maximum pressure of 414 MPa using an AutoPore IV 9510 fully automated mercury manometer in Shanghai, China.

Interfacial transition zone analysis was performed on concrete test samples by backscattering electron tests (BSE). Firstly, test pieces with a thickness of about 3–5 mm were cut from the concrete specimens. When cutting, the concrete pieces were cut parallel to the surface of the specimen with a cutter at 15 mm and 18–20 mm from the surface of the specimen, respectively. Then, core samples (including aggregates) were drilled on the cut surface of the concrete test blocks using an electric drill and hollow core bit (8–14 mm inner diameter of the drill bit). After sampling, the samples were soaked in anhydrous ethanol for 7 days to terminate hydration, and then dried for 3 days (drying temperature of 50 ± 2 °C). The dried samples were immersed in epoxy resin for 24 h. The sample to be tested is then prepared by grinding, shaping, ultrasonic cleaning and drying (drying temperature of 50 ± 2 °C). The magnification of the test image is 500 times, the resolution is 1024 × 768 pixels. Image J 1.8.0 image processing software was used to perform quantitative analysis and calculations on the images.

## 3. Results and Discussion

### 3.1. Mechanical Properties of ISD System 

#### 3.1.1. Effect of Steel Slag Doping Ratio on Mechanical Properties 

**Figure 2:**
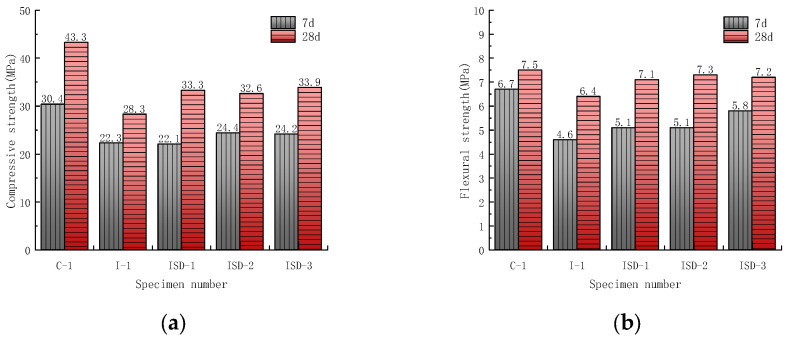
Effect of steel slag doping ratio on (**a**) compressive strength and (**b**) flexural strength of the ISD admixture.

In order to clarify the effect of steel slag admixture on the strength of the ISD system, three cement mortar specimens with 1:3, 1:1 and 3:1 steel slag–desulfurization ash ratios were prepared, and the specimens were maintained for 7 d and 28 d before strength testing. The test results are shown in Figure 2.

The results in Figure 2 show that although the 7 d and 28 d compressive strength of the ISD admixture system is lower than that of the pure cement system, it is significantly higher than that of the iron tailings admixture system, and the flexural strength does not vary significantly. This is attributed to the fact that CaO in the desulfurization ash (Table 1) can react with SiO_2_ in the iron tailings in a volcanic ash reaction to form CSH [30]. In addition, steel slag contains more C_2_S and C_3_S, which can provide cementing materials, such as Ca(OH)_2_ and C-S-H, for the iron tailing dopant system [31,32]. More interestingly, the preliminary compressive strength of the ISD admixture system increases and then decreases as the steel slag admixture ratio increases. Compared with CaO-rich desulfurization ash (Table 1), steel slag contains more C_2_S and C_3_S, which can make the ISD system produce more gelling materials, such as C-S-H 32. However, the continued substitution of steel slag for desulfurization ash will cause a reduction in the pre-strength of ISD. This is due to the inhibition of the early hydration of the cement by steel slag, and the higher the amount of steel slag, the more obvious the effect [33]. The late compressive strength of the ISD system shows an overall increasing trend with the increase in the steel slag admixture. This is mainly because the steel slag will improve the later hydration conditions of the cement by increasing the actual water–cement ratio and the nucleation of the inert phase, etc., and promote the later hydration of the cement [34].

#### 3.1.2. Effect of Iron Tailings Doping Ratio on Mechanical Properties

**Figure 3:**
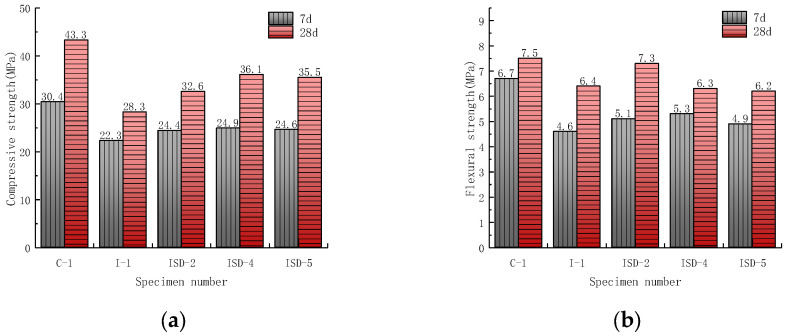
Effect of iron tailings doping ratio on (**a**) compressive strength and (**b**) flexural strength of the ISD admixture.

To further clarify the effect of iron tailings doping ratio on the strength of the ISD admixture, the test was set up by 1:1, 1:2 and 1:4 of three kinds of iron tailing and steel slag–desulfurization ash ratio doping cement preparation of the colloidal sand specimens, and the strength test was carried out after 7 d and 28 d of specimen maintenance. The test results are shown in Figure 3.

The compressive strength of the ISD system gradually increased with the increase in the iron tailings admixture, which is contrary to the results of Cheng et al. [35]. This can be attributed to two aspects: (1) iron tailings are relatively fine particles with good microfilling effect [11,36]. The filling effect makes the overall structure of the ISD system denser. (2) Iron tailings have some nucleation effect [18]. Nucleation improves the hydration condition of cement and promotes the hydration of cement. However, the 7 d and 28 d compressive strength of the ISD system slightly decreased when the iron tailings incorporation ratio continued to increase. This is similar to the results of Wu et al. [37]. It is mainly due to the relatively low activity of iron tailings [2]. When the amount of iron tailings blended is too high, the strength loss brought to the ISD system by the low activity of iron tailings is greater than the strength supplement brought to the ISD system by iron tailings through filling and nucleation effect. Therefore, the strength of the ISD system will decrease if the iron tailing continues to increase.

### 3.2. Effect of Chemical Activator on the ISD System

#### 3.2.1. Mechanical Properties 

**Figure 4:**
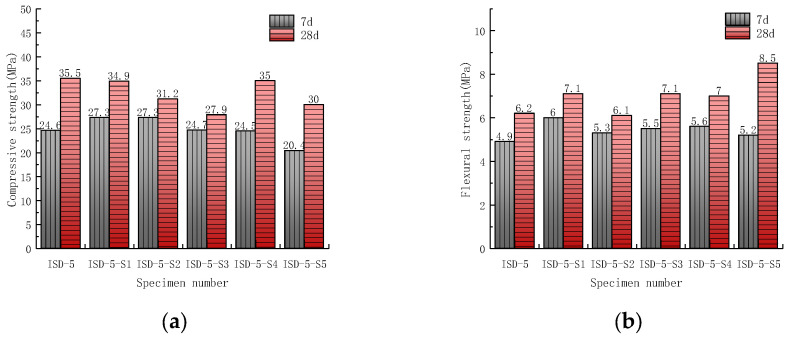
Effect of activator Na_2_SiO_3_ on the (**a**) compressive strength and (**b**) flexural strength of the ISD admixture.

On the basis of iron tailings–steel slag–desulfurization ash doping ratio of 1:2:2, an attempt was made to further investigate the effect of an Na_2_SiO_3_ activator on the strength of the ISD admixture. Five Na_2_SiO_3_ doping amounts (0.4%, 0.6%, 0.8%, 1.0% and 1.2%, respectively) of ISD admixture sand specimens were prepared, and the strength test was carried out after 7 d and 28 d of specimen maintenance. The test results are shown in Figure 4.

Na_2_SiO_3_ will react with Ca(OH)_2_ to form C-S-H and NaOH [38,39], and NaOH can provide a higher alkaline environment for the ISD system. In a certain alkalinity range, the higher alkalinity is favorable to the breakage of silica–oxygen and aluminum–oxygen bonds in the solid waste [40], which promotes more solid waste for volcanic ash reaction, and Na_2_SiO_3_ will also promote the rapid hydration reaction of C_3_A in the ISD system, so Na_2_SiO_3_ can promote the pre-strength growth of the ISD system. However, with the gradual increase in Na_2_SiO_3_ incorporation, more Na_2_SiO_3_ reacts with Ca(OH)_2_ to generate C-S-H, causing many cementitious materials in the ISD system to wrap around the surface of solid waste and cement particles, which inhibits the hydration of cement and the volcanic ash effect of solid waste; in addition, Na_2_SiO_3_ will cause the porosity of the ISD system to rise, thus reducing the overall density of the ISD system [38]. Therefore, excessive Na_2_SiO_3_ will not only inhibit the late compressive strength of the ISD system, but also the early strength of the ISD system.

#### 3.2.2. Thermal Analysis 

TG-DTA can be used to analyze the generation of gel water and Ca(OH)_2_ in the gelling material products, to realize the quantitative analysis of the degree of volcanic ash reaction in the ISD system, and then to determine the mechanism of the action of Na_2_SiO_3_ activator on the mechanical properties of the ISD admixture system.

**Figure 5:**
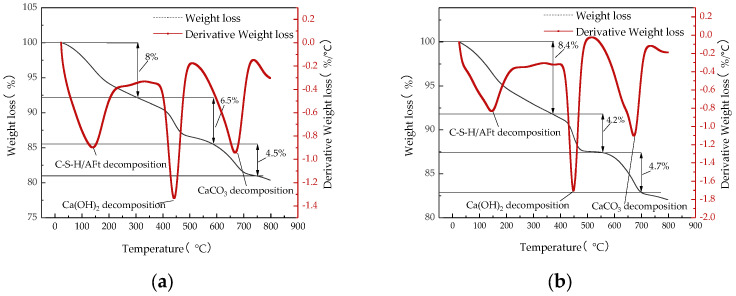

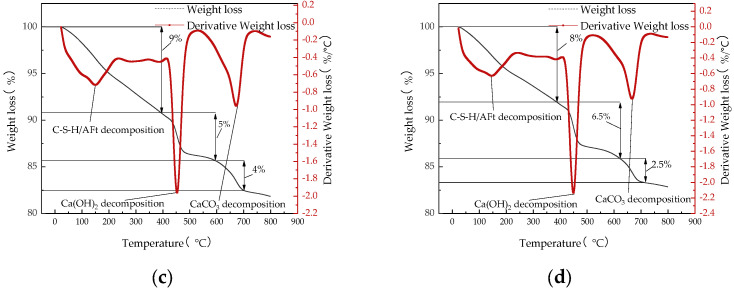
This TG-DTA curve is with respect to (**a**) 7 d Na_2_SiO_3_/0g, (**b**) 7 d Na_2_SiO_3_/0.81g, (**c**) 28 d Na_2_SiO_3_/0 g, and (**d**) 28 d Na_2_SiO_3_/0.81 g.

In the experiment, iron tailings: steel slag: desulfurization ash = 1:2:2, the ratio of the total mass of solid waste admixture to the total mass of cement is 3:7, the water-to-binder ratio is 0.5, the ratio of binder-to-sand is 1:3 (mass ratio), the activator is 0% and 0.6% doping of Na_2_SiO_3_, respectively, after 7 d and 28 d of standard maintenance for comparative analysis; the experimental results are represented by Figure 5.

Figure 5 shows that the regular characteristics of the specimens at each hydration age with different Na_2_SiO_3_ doping are similar, with three heat losses occurring in three heat absorption peaks for each specimen. The first occurred at about 100 °C, when the C-S-H and Aft dehydroxylation of the gel water mainly occurred; the second occurred at about 450 °C, when the dehydroxylation of Ca(OH)_2_ followed by water loss mainly occurred; and the third occurred at about 670 °C, when the decomposition of calcium carbonate mainly occurred [41]. The gel water and Ca(OH)_2_ contents of 7 d and 28 d ISD admixture specimens determined by TG-DTA microanalysis are represented by Table 5.

Table 5 shows that the Ca(OH)_2_ content in the reaction of the 7 d ISD system without Na_2_SiO_3_ is greater than that of the ISD system with Na_2_SiO_3_, while the gel water content is less than that of the ISD system with Na_2_SiO_3_, and the experimental results of Ca(OH)_2_ and gel water content fully demonstrate that Na_2_SiO_3_ improves the precompressive force of the ISD system by reacting with Ca(OH)_2_ to produce gels, such as C-S-H. The Ca(OH)_2_ content in the reaction of the 28 d ISD system without Na_2_SiO_3_ is lower than that of the ISD system with Na_2_SiO_3_, while the gel water content is greater than that of the ISD system with Na_2_SiO_3_, which fully indicates that due to the reaction of a large amount of Na_2_SiO_3_ with Ca(OH)_2_ to generate C-S-H, which causes many cementitious materials in the ISD system to wrap around the surface of solid waste and cement particles, inhibiting the hydration of cement and the volcanic ash effect of solid waste, thus reducing the late strength of the ISD system.

### 3.3. Compressive Strength of ISD Admixture Concrete 

**Figure 6:**
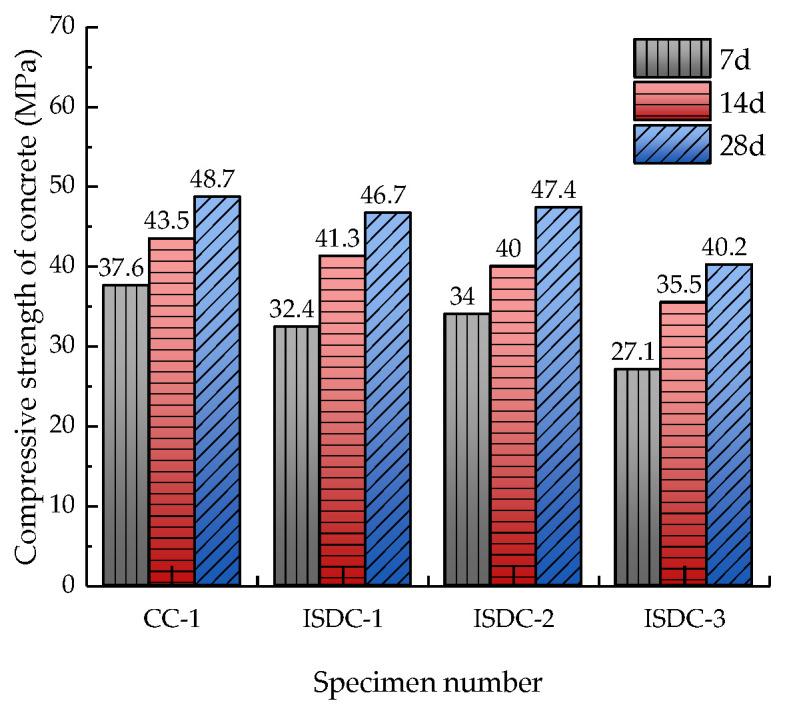
Compressive strength of concrete with different ISD admixtures.

In order to test the effect of the ISD admixture in concrete and to determine the effect of ISD admixture dosage on the compressive strength of concrete, in the experiment, four ISD doping amounts of 0, 10%, 20% and 30% (indicated by CC-1, ISDC-1, ISDC-2 and ISDC-3, respectively) were set; the water–cement ratio was 0.44, and the doping ratio was iron tailings:steel slag:desulfurization ash = 1:2:2, and the comparative analysis was carried out after 7 d, 14 d and 28 d of standard maintenance. The experimental results are represented by Figure 6.

Figure 6 shows that the 7 d compressive strength of ISD admixture concrete is significantly lower than that of cement concrete, but the 28 d compressive strength is not significantly different from that of cement concrete. The 7 d, 14 d and 28 d compressive strength of ISD admixture concrete decreases with increasing admixture amount. ISD admixture has no effect on the late strength of concrete, but causes the early strength to be reduced. An analysis of the reason shows, from the chemical composition Table 1, it can be seen that steel slag and desulfurization ash mainly contain CaO, and iron tailings mainly contain SiO_2_, which leads to the ISD admixture mainly causing a volcanic ash reaction, but the volcanic ash reaction rate is much lower than the hydrolytic hydration reaction of cement [42], thus causing the early strength of the ISD admixture concrete to be insufficient. Plus, with the increase in the curing time, the volcanic ash reaction and hydrolytic hydration reaction of the ISD admixture concrete was carried out sufficiently, so that the later strength of the ISD admixture concrete was not affected. In ISD admixture concrete, with the increase in admixture, the amount of cement admixture in concrete decreased, which led to the decrease in hydration reaction in concrete. While more volcanic ash reaction replaced the hydration reaction, volcanic ash reaction provided much less cementitious material than the hydration reaction, which led to the decrease in compressive strength of the ISD admixture concrete with the increase in admixture.

### 3.4. Compressive Mercury Analysis 

**Figure 7:**
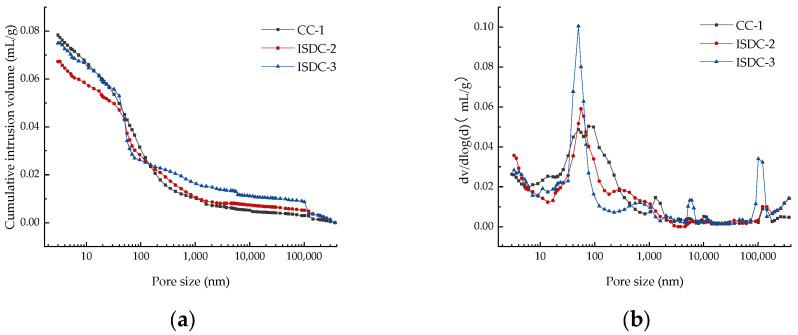

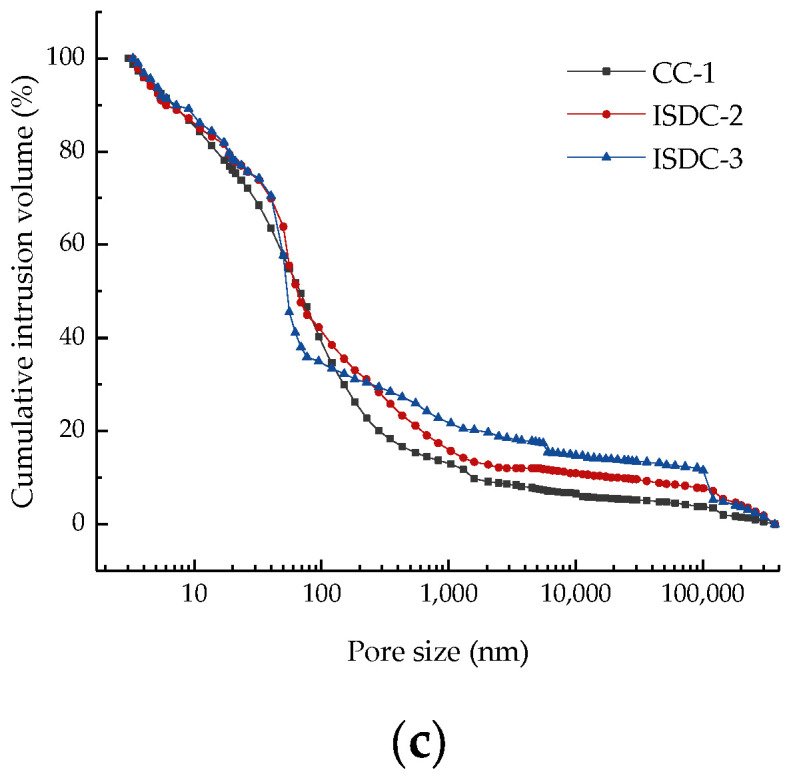
(**a**) Cumulative pore volume, (**b**) pore distribution, and (**c**) cumulative pore ratio of concrete at each ISD dose.

In order to further explain the mechanism of the effect of the variation of the ISD admixture on the compressive strength of concrete in the direction of total and harmful pore volume, the concrete with three ISD admixture levels of 0, 20% and 30% and cured for 28 d in Section 2.3 was analyzed in a mercury compression test. The experimental results are represented by Figure 7 and Table 6.

Table 6 is a summary of the results of the piezometric tests, and it can be seen from Table 6 that the percentage of multiharmful and harmful pores in the ISD admixture concrete is lower than that of cement concrete, which indicates the filling effect of the ISD admixture on the concrete. However, the 28 compressive strength of ISD-doped concrete in Section 2.3 is slightly lower than that of cement concrete, and the strength of concrete is mainly influenced by the activity effect and microfilling effect of cementitious materials [43,44], which indicates that ISD admixtures provide a microfilling effect for concrete, but at the same time cause a reduction in the activity effect of concrete. Moreover, from Table 6 it can be seen that with the increase in the ISD admixture, the percentage of the total number of multiharmful and harmful pores in ISD concrete decreases, while the total pore volume increases. The above test results and the test results of 28 d compressive strength reduction of ISD concrete in Section 2.3 show that the strength of ISD concrete is influenced by the total pore volume. As the amount of ISD admixture increases, the total pore volume of the ISD concrete also increases.

### 3.5. Microstructure Analysis 

**Figure 8:**
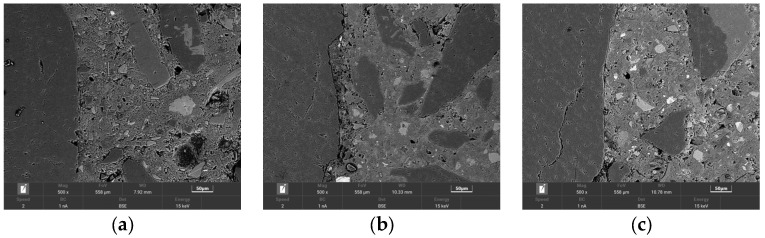
28 d backscatter analysis of (**a**) CC-1, (**b**) ISDC-2, (**c**) ISDC-3 concrete.

**Figure 9:**
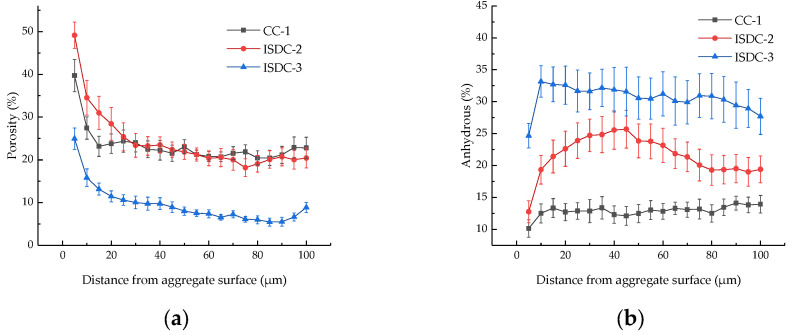
(**a**) The 28 d interfacial transition zone pore distribution and (**b**) 28 d interfacial transition zone non-hydration distribution of ISD-doped concrete.

The interfacial transition zone is a weak region of concrete, which has a large influence on the mechanical properties of concrete [45]. In order to study the effect of the variation of ISD admixture on the properties of the interfacial transition zone of concrete, and then to clarify the mechanism of the effect of the ISD admixture on the mechanical properties of concrete, the backscattering technique was analyzed for concrete with three ISD admixtures of 0, 20%, and 30% and cured for 28 d in Section 2.3. The experimental results are represented by Figure 8 and Figure 9.

It can be seen from Figure 8 that various unhydrated particles and various micropores are present in the transition zone of the concrete interface. The ISD-doped concrete has a denser structure, fewer pores and smaller pore size compared to cement concrete. To make clearer the effect of the ISD admixture on the number of pores and unhydrated material in concrete, the distribution of the number of pores in the interfacial transition zone shown in Figure 9a and the distribution of the number of unhydrated materials in the interfacial transition zone shown in Figure 9b were plotted by a dozen BSE plots. It can be seen from Figure 9a that the porosity of 20% ISD-doped concrete is not much different from that of cement concrete, but both are significantly higher than that of 30% ISD-doped concrete, which further confirms that the ISD admixture has a filling effect on concrete, especially on the interface transition zone it is more obvious. Figure 9b shows that the unhydrated particles in the interfacial transition zone of ISD-doped concrete are significantly higher than that of cement concrete, which further confirms that ISD provides a filling effect to concrete while causing loss of concrete activity.

The BSE test results further confirm the results of the mercury compression test in Section 3.4, that although ISD provides a filling effect to the concrete, it also causes a reduction in the active reactants of the concrete. This shows that the combined effect of the microfilling effect of ISD and the reactive effect affects the strength development of ISD concrete.

## 4. Conclusions

In this study, a ternary solid waste admixture was prepared using iron tailings, steel slag and desulfurization ash and its application to concrete was verified. The effects of dopant type and chemical activator on the mechanical properties of ISD were investigated, and the mechanism of chemical activators on ISD was analyzed by TG-DTA. The strength, porosity, and interfacial transition zone of ISD concrete were tested by concrete compressive test, MIP, and BSE. This study provides a scientific basis for the preparation of ISD dopants and promotes the consumption of bulk solid waste iron tailings. The main test findings are as follows:

1. The 7 d and 28 d compressive strengths of an ISD admixture are significantly higher than those of an iron tailings single admixture. With the increase in the proportion of steel slag admixture, the pre-strength of the ISD admixture grows first and then decreases. The 7 d and 28 d compressive strengths of the ISD admixture showed a trend of increasing and then decreasing with the increase in the proportion of iron tailings. The 7 d and 28 d compressive strengths of the ISD system reached 24.9 MPa and 36.1 MPa, respectively, when the ratio of iron tailings:steel slag:desulfurization ash = 1:1:1.
2. The appropriate amount of Na_2_SiO_3_ can promote the early strength growth of an ISD admixture and also inhibit the later strength of an ISD admixture. Na_2_SiO_3_ is suitable for the early strength agent of an ISD admixture, but the amount of admixture should not exceed 0.6% of the admixture. Na_2_SiO_3_ is used to improve the early strength of the ISD system by promoting the consumption of Ca(OH)_2_ in the ISD system to produce C-S-H. However, in the late reaction of the ISD system, Na_2_SiO_3_ inhibits the late strength development of the ISD system by inhibiting Ca(OH)_2_ production.
3. An ISD admixture will cause the early strength of concrete to decrease. The late strength of concrete can also be reduced when ISD is mixed in excessive amounts. Concrete with ISD dosing of 30% or less meets C40 requirements.
4. The strength of ISD concrete is influenced by the total pore volume, and the total pore volume of ISD concrete increases with the increase in ISD admixture. ISD will provide a filler effect to concrete, but it will also cause a reduction in the active reactants of concrete, and the combined effect of a microfill effect and an active effect affects the strength development of ISD concrete.

## 5. Recommendations

In this paper, although the compressive strength of concrete with ISD admixture of 30% or less meets the C40 requirement, the mechanical properties of ISD mixes need to be further investigated, as follows:

1. Since the water-to-cement ratio can seriously affect the workability of cement mortar and the performance of concrete, further studies on the effect of water-to-cement ratio on the performance of ISD concrete are needed.
2. If increasing the amount of ISD in concrete will further improve the economic value of ISD concrete, it is necessary to further investigate the performance of high-dosage ISD concrete.
3. Durability is an important property of concrete, and it is necessary to study the durability of ISD concrete, such as impermeability and resistance to sulfate attack.
4. Admixtures can significantly improve the performance of concrete, and further research is needed to investigate the admixtures suitable for ISD concrete.

## Figures and Tables

**Table 1:** Chemical composition of raw materials/%.

Ingredients	Iron Tailings	Steel Slag	Desulfurization Ash
SiO_2_	66.26	15.20	1.47
Al_2_O_3_	4.78	2.53	1.14
Fe_2_O_3_	14.37	27.54	0.44
MgO	6.33	6.05	2.00
CaO	7.77	42.65	93.85
SO_3_	0.48	0.12	0.19

**Table 2:** ISD system material mix proportion.

Number	Raw Materials	Cement Replacement Rate and Proportioning	Solid Waste/g	Cement/g	Standard Sand/g	Water/g
C	Cement	100%	0	450	1350	225
I-1	Iron Tailings	30%	135	315		
ISD-1	Iron Tailings–Steel Slag–Desulfurization Ash	30% (4:1:3)				
ISD-2	Iron Tailings–Steel Slag–Desulfurization Ash	30% (2:1:1)				
ISD-3	Iron Tailings–Steel Slag–Desulfurization Ash	30% (4:3:1)				
ISD-4	Iron Tailings–Steel Slag–Desulfurization Ash	30% (1:1:1)				
ISD-5	Iron Tailings–Steel Slag–Desulfurization Ash	30% (1:2:2)				

**Table 3:** Experimental study on the effect of variation of activator Na_2_SiO_3_ doping on the mechanical properties of ISD dopant system/g.

Number	Raw Materials	Cement Replacement Rate and Proportioning	Na_2_SiO_3_ Doping/%	Solid Waste/g	Cement/g	Standard Sand/g	Water/g
ISD-5	Iron Tailings–Steel Slag–Desulfurization Ash	30% (1:2:2)	0				
ISD-5-S1	Iron Tailings–Steel Slag–Desulfurization Ash	30% (1:2:2)	0.4	135	315	1350	225
ISD-5-S2	Iron Tailings–Steel Slag–Desulfurization Ash	30% (1:2:2)	0.6				
ISD-5-S3	Iron Tailings–Steel Slag–Desulfurization Ash	30% (1:2:2)	0.8				
ISD-5-S4	Iron Tailings–Steel Slag–Desulfurization Ash	30% (1:2:2)	1.0				
ISD-5-S5	Iron Tailings–Steel Slag–Desulfurization Ash	30% (1:2:2)	1.2				

**Table 4:** Experimental study on the effect of ISD admixture variation on concrete/g.

Number	Raw Materials	Cement Replacement Rate and Proportioning	Cement/g	Iron Tailing Waste Rock/g	Iron Tailing Sand/g	Water Reducing Agent/g	Water/g
CC-1	Iron Tailings–Steel Slag–Desulfurization Ash	0% (1:2:2)	420	1110	740	4.5	185
ISDC-1	Iron Tailings–Steel Slag–Desulfurization Ash	10% (1:2:2)	378				
ISDC-2	Iron Tailings–Steel Slag–Desulfurization Ash	20% (1:2:2)	336				
ISDC-3	Iron Tailings–Steel Slag–Desulfurization Ash	30% (1:2:2)	294				

**Table 5:** Gel water and Ca(OH)_2_ content of ISD systems.

Number	Age/d	H_2_O/mg	Ca(OH)_2_/mg
ISD-5	7	0.8	2.67
ISD-5-S2	7	0.84	1.72
ISD-5	28	0.9	2.05
ISD-5-S2	28	0.8	2.67

**Table 6:** Results of mercury intrusion porosimetry.

Number	Total Pore Volume (mL/g)	The Most Countable Pore Size (nm)	Harmless Hole (%)	Less Harmful Holes (%)	Harmful Holes (%)	Multi-Hazardous Holes (%)
CC-1	0.07835	77.1	24.67	17.73	34.8	22.8
ISDC-2	0.06730	55.3	22.98	21.5	27.14	28.38
ISDC-3	0.07506	40.26	22.93	31.54	16.09	29.44
